# Breviscapine reduces acute lung injury induced by left heart ischemic reperfusion in rats by inhibiting the expression of ICAM-1 and IL-18

**DOI:** 10.3892/etm.2013.1287

**Published:** 2013-09-04

**Authors:** YUXIA WANG, MINGLI JI, LIPING CHEN, XINJUN WU, LEI WANG

**Affiliations:** 1 Department of Pathophysiology, Xinxiang Medical University, Xinxiang, Henan 453003;; 2 Department of General Surgery, The First Affiliated Hospital of Xinxiang Medical University, Weihui, Henan 453100, P.R. China

**Keywords:** breviscapine, left heart ischemic reperfusion, acute lung injury, ICAM-1, IL-18

## Abstract

It has been demonstrated that breviscapine is able to treat coronary disease and reduce the inflammatory response; however, there are no relevant reports concerning its effects on the expression of inflammatory factors in acute lung injury induced by left heart ischemic reperfusion and the underlying mechanisms. In this study, we created a left heart ischemia-reperfusion model in rats to investigate the effects of breviscapine on the expression of interleukin 18 (IL-18) and intercellular adhesion molecule-1 (ICAM-1), as well as to determine the possible mechanisms involved in the protective effects of breviscapine on respiratory function. The left heart ischemia-reperfusion model was created by ligating the anterior descending branch of the coronary artery for 30 mins followed by reperfusion. Rats in the treatment group (TG) were treated with breviscapine (10 mg/kg) and the rats in the control group (CG) received normal saline. Ten rats in the two groups were sacrificed at three points: 30 min after ligating (T1), 30 min after reperfusion (T2) and 60 min after reperfusion (T3). A respiration curve was produced and the arterial partial pressure of oxygen (PaO_2_) was measured for all rats. Additionally, the expression levels of IL-18 and ICAM-1 were determined and the correlation between IL-18 and ICAM-1 expression in lung tissue was analyzed. The level of IL-18 in peripheral blood and bronchialalveolar lavage fluid (BALF) was also measured. The respiration amplitude was lower and the duration time was shorter in the TG rats than in the CG rats at T1, T2 and T3. The expression levels of IL-18 and ICAM-1 in the TG group were clearly reduced. The level of IL-18 in the peripheral blood and BALF was downregulated following the administration of breviscapine. These results demonstrate that breviscapine inhibits the expression of IL-18 and ICAM-1, thereby protecting the lungs from inflammatory cascade responses.

## Introduction

Symptoms and signs of acute respiratory insufficiency, including cough, expectoration and asthma, may occur during thrombolytic therapy of left ventricular myocardial infarction, and may cause respiratory failure. Therefore, protecting the lungs from injury throughout thrombolytic therapy is becoming a focus of particular interest in cardiovascular research.

*Erigeron breviscapus* is a wild herbal plant of Yunnan Province, China. The major effective component is a flavone, breviscapine. Breviscapine activates blood circulation, removes blood stasis, relieves pain and enforces microcirculation. The pharmacological effects of breviscapine include the inhibition of platelet and erythrocyte adhesion, reduction of blood viscosity and dilation of blood vessels (1,2). Breviscapine is commonly used in the clinic for treating coronary heart disease. Previous studies (3,4) have identified that breviscapine suppresses the production of procalcitonin (PCT) and neutrophil elastase (NE), reduces the systemic inflammatory response and protects the lungs of children undergoing open heart surgery. Another study (5) showed that a breviscapine injection had anti-injury effects on hypoxic-ischemic brain damage in neonatal rats, possibly by reducing the expression of Bcl-2 and Bax.

Since breviscapine may be used to treat coronary heart disease and reduce the systemic inflammatory response, it is possible that breviscapine may protect the lungs in left heart ischemic reperfusion. There are no relevant reports of whether breviscapine affects the expression of inflammatory factors in acute lung injury induced by left heart ischemic reperfusion, or concerning the underlying mechanism.

In the present study, an acute lung injury rat model induced by left heart ischemic reperfusion was established and treated with breviscapine. We investigated the effect of breviscapine on the expression of interleukin 18 (IL-18) and intercellular adhesion molecule-1 (ICAM-1) and provide a possible mechanism of the protective role of breviscapine on respiratory function. The results revealed that breviscapine decreased the expression of IL-18 and ICAM-1, and reduced inflammatory injury in the lungs. The present study provides a theoretical basis for the clinical application of breviscapine to treat left ventricular dysfunction in acute respiratory failure.

## Materials and methods

### 

#### Laboratory animals

Sixty healthy rats of mixed gender, weighing 250–350 g were obtained from the Experimental Animal Center of Zhengzhou University (Zhengzhou, China) and were randomly divided into two groups: the treatment group (TG; n=30) and the control group (CG; n=30). The rats in the TG received breviscapine and the rats in the CG received normal saline. The study was approved by Ethics Committee of Xinxiang Medical University (Xinxiang, China). The research was compliance with the principles enunciated in Helsinki ethical principles declaration.

#### Main reagents and instruments

Breviscapine injections were purchased from Heilongjiang Feixia Pharmaceutical Industry Co., Ltd. (Harbin, China). The BL-420 biological signal collecting and processing system (Chengdu TME Technology Co., Ltd., Chengdu, China) was supplied by the Functional Laboratory of Xinxiang Medical University. A blood gas analyzer was purchased from Shanghai Yuyan Instruments Co., Ltd. (Shanghai, China). IL-18 and ICAM-1 polyclonal antibodies, and an SP kit were purchased from Beijing Zhong Shan -Golden Bridge Biological Technology Co., Ltd. (Beijing, China). A myeloperoxidase kit was supplied by Nanjing Jiancheng Bioengineering Institute (Nanjing, China).

### Methods

#### Surgery

Surgery was performed according to a previously described method (6). Briefly, a cannula was inserted into the left external jugular vein after the rats had received general anesthesia with 4% chloral hydrate (1 ml/100 g body weight). Each rat underwent thoracotomy and left anterior descending coronary artery ligation. Rats in the TG received treatment with a breviscapine injection (10 mg/kg body weight) through the external jugular vein cannula once the left anterior descending coronary artery had been ligated for 10 min. The rats in the CG received normal saline. The rats in the two groups were ligated for 30 min and then reperfused.

In each rat, the diaphragm was exposed prior to thoracic surgery and connected to the BL-420 biological signal collecting and processing system through a needle electrode. Then, respiratory curve data was obtained. Following the collection of 5 ml peripheral blood samples at 30 min after ligaturing (T1), 30 min after reperfusion (T2) and 60 min after reperfusion (T3), and the measurement of the arterial partial pressure of oxygen (PaO_2_) using a blood gas analyzer, all rats were sacrificed. The lungs were removed and bronchialalveolar lavage was performed immediately. Then, 5 ml bronchialalveolar lavage fluid (BALF) was collected. The lower lobe of the right lung was removed and fixed with paraformaldehyde solution (1 ml/100 g).

*Immunohistochemistry.* Formalin-fixed and paraffin-embedded lung tissues were deparaffinized and rehydrated, quenched with 3% H_2_O_2_, blocked with 5% normal goat serum and probed with rabbit anti-rat IL-18/ICAM-1 antibody. Detection was with biotinylated anti-rabbit IgG, followed by incubation with avidin-biotin complex and substrate (diaminobenzidine) followed with hematoxylin counterstaining. These experiments were performed in triplicate.

*Enzyme-linked immunosorbent assay (ELISA).* The IL-18 levels in serum and BALF were measured by ELISA according to the manufacturer’s instructions (Nanjing Huadong electron group medical equipment Co., Ltd., Nanjing, China), the sensitivity and specificity of the method are 99 and 92%, respectively. Each diluted sample (100 *μ*l) was applied in triplicate on 96-well plates pre-coated with capture antibody for 2 h, followed by incubation with detection antibody for 1 h and avidin-HRP for 30 min. The plates were then developed using 3,3′,5,5′-tetramethylbenzidine and terminated by 2 M H_2_SO_4_. The OD value was then recorded at 450 nm. These experiments were performed in triplicate.

#### Statistical analysis

All data were analyzed using SPSS 11.0 statistical software (SPSS, Inc., Chicago, IL, USA). Respiration curve data and the level of IL-18 in peripheral blood and BALF were analyzed by analysis of variance (ANOVA). The rank sum test and Spearman’s rank correlation analysis were used to analyze the expression of IL-18 and ICAM-1 in the two groups. P<0.05 was considered to indicate a statistically significant difference.

## Results

### 

#### Comparison of the respiration curves of the two groups

**Figure 1.**
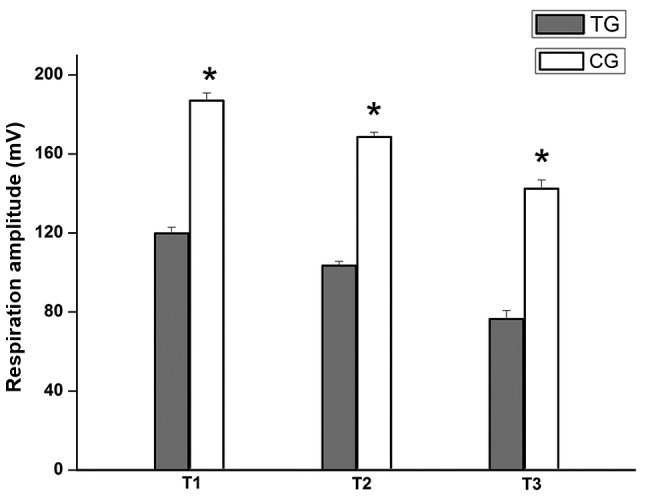
Comparison of respiration amplitude between the two groups. According to the BL-420 biological signal collecting and processing system, the respiration amplitude of TG rats was lower at the same time-point compared with that of CG rats. ^*^P<0.05, vs. TG rats. TG, treatment group; CG, control group.

**Figure 2.**
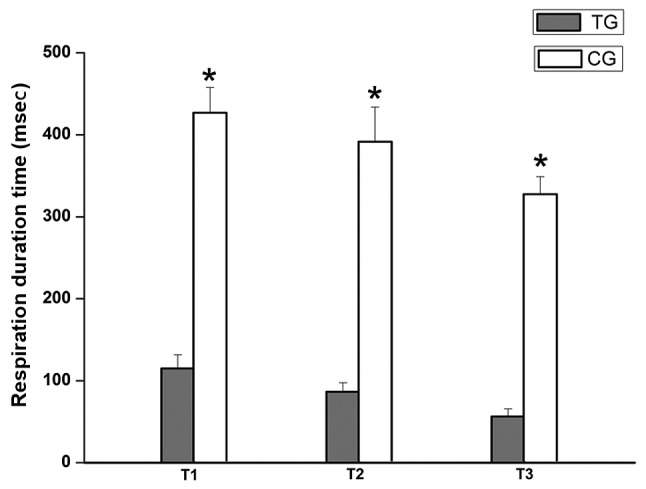
Comparison of respiration duration time between the two groups. The results of the BL-420 biological signal collecting and processing system revealed that the duration time of respiration of the TG rats was shorter at the same time-point compared with that of the TG rats. ^*^P<0.001, vs. TG rats. TG, treatment group; CG, control group.

A respiration curve was collected and recorded using the BL-420 biological signal collecting and processing system. Respiration amplitude reflects alveolar ventilation; respiration amplitude is likely to deepen or enlarge when the lung function reduces. Respiration duration time reflects the breathing rate. As shown in Figs. 1 and 2, at the same time-point, the respiration amplitude of TG rats was lower and the duration time was shorter compared with the respective value in CG rats. The respiration curve shows that after using breviscapine, the respiration curve of TG rats tended to be closer to normal compared with that of CG rats. Therefore, breviscapine may enhance respiratory function.

#### Comparison of PaO_2_ between the two groups

**Figure 3.**
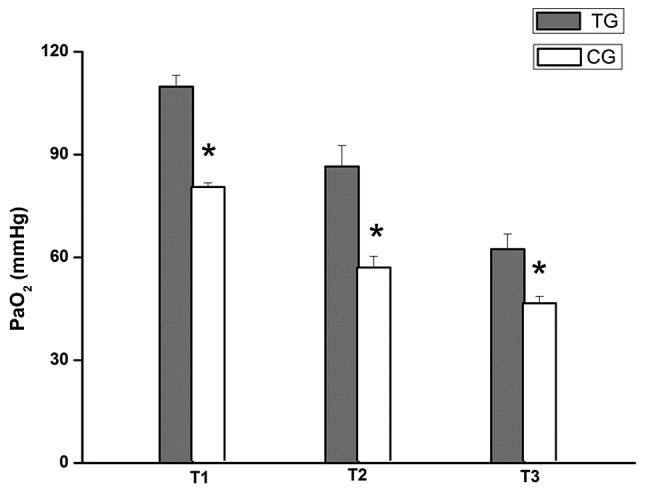
Comparison of PaO_2_ between the two groups. PaO_2_ was measured at the indicated time-points using a blood gas analyzer. PaO_2_ in the TG was higher compared with that in the CG at T1, T2 and T3. ^*^P<0.001 vs. TG rats. TG, treatment group; CG, control group; T1, 30 min after ligating; T2, 30 min after reperfusion; T3, 60 min after reperfusion; PaO2, arterial partial pressure of oxygen.

PaO_2_ was measured with a blood gas analyzer. PaO_2_ is used to judge respiratory function. PaO_2_ reduces during respiratory dysfunction. As shown in Fig. 3, PaO_2_ in the TG was higher compared with that in the CG, which suggests that respiratory function was improved following the administration of breviscapine.

#### Comparison of the expression of IL-18 in lung tissue between the two groups

**Figure 4.**
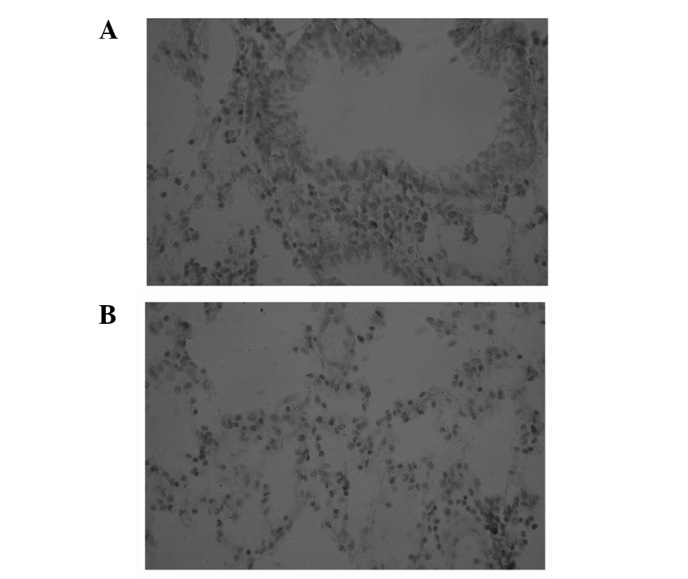
Comparison of the expression of IL-18 in lung tissue (immunohistochemical staining; magnification, ×400). (A) Expression of IL-18 in lung tissue at T2 in TG rats. (B) Expression of IL-18 in lung tissue at T2 in CG rats. The levels of IL-18 protein in the TG were clearly lower compared with those in the CG at T1, T2 and T3. T1, 30 min after ligating; T2, 30 min after reperfusion; T3, 60 min after reperfusion; TG, treatment group; CG, control group; IL-18, interleukin 18.

IL-18 is pro-inflammatory factor, which is released as a result of inflammation. As IL-18 levels increase, more pro-inflammatory cytokines are released; therefore, inflammation is amplified. Immunohistochemistry was used to examine the expression of IL-18. The immune response products of IL-18 are mainly located in the cytoplasm. As shown Table I and Fig. 4, the positive expression of IL-18 in TG lung tissue was lower compared with that in CG lung tissue, which indicates that acute inflammation in TG lung tissue was milder.

#### Comparison of the expression of ICAM-1 in lung tissue between the two groups

**Figure 5.**
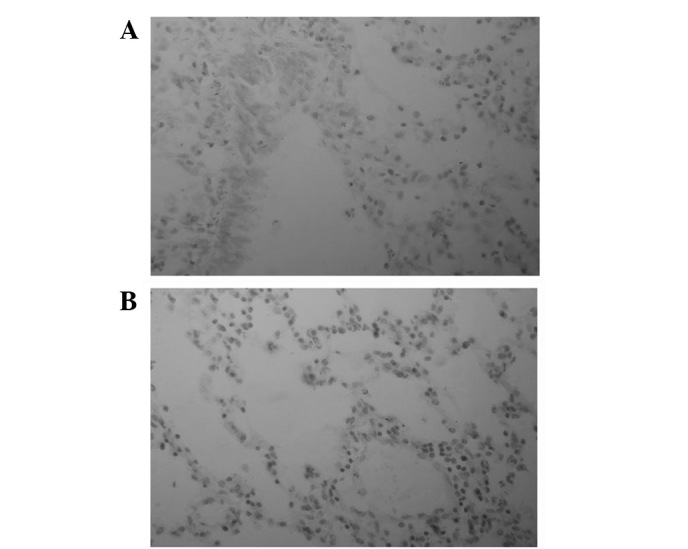
Comparison of the expression of ICAM-1 (immunohistochemical staining; magnification, ×400). (A) Expression of ICAM-1 in lung tissue at T2 in TG rats. (B) Expression of ICAM-1 in lung tissue at T2 in CG rats. The level of positive expression of ICAM-1 protein in the TG was lower compared with that in the CG at T1, T2 and T3. T1, 30 min after ligating; T2, 30 min after reperfusion; T3, 60 min after reperfusion; TG, treatment group; CG, control group; ICAM-1, inflammatory cell adhesion molecule-1.

The immune response products of ICAM-1 were mainly located in the cytoplasm or membrane. The positive expression of ICAM-1 was lower in the TG compared with that in the CG (Table II and Fig. 5), which demonstrates that breviscapine attenuates the damage of lung tissue.

#### Correlation between IL-18 and ICAM-1 expression in lung tissue of the TG

In order to further evaluate whether the expression of IL-18 is correlated with the expression of ICAM-1, we used the rank sum test and Spearman’s rank correlation analysis. The results demonstrated that the expression of IL-18 and ICAM-1 were directly correlated (Table III).

#### Comparison of the level of IL-18 in peripheral blood and BALF between the two groups

**Figure 6.**
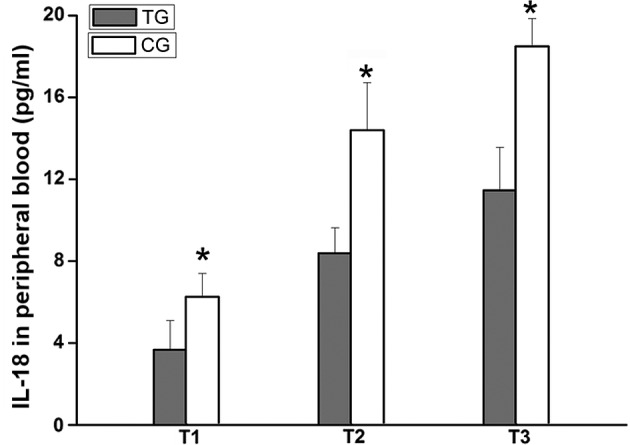
Comparison of the level of IL-18 in peripheral blood between the two groups (n=10). The level of IL-18 in the peripheral blood of TG rats was lower compared with that of CG rats at T1, T2 and T3. ^*^P<0.001, vs. TG rats. T1, 30 min after ligating; T2, 30 min after reperfusion; T3, 60 min after reperfusion; TG, treatment group; CG, control group; IL-18, interleukin 18.

**Figure 7.**
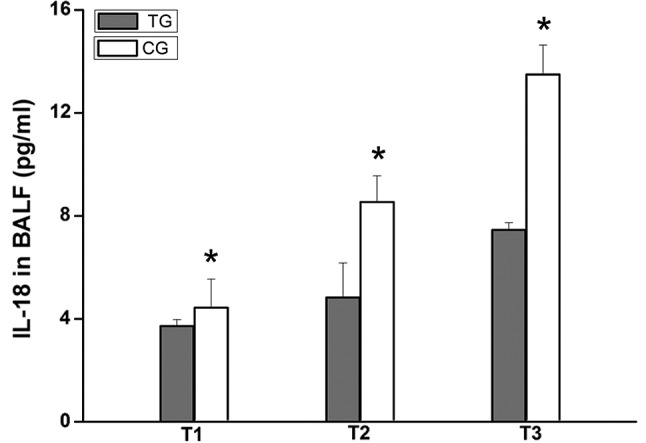
Comparison of the level of IL-18 in BALF between the two groups (n=10). The level of IL-18 in BALF was assessed using ELISA. The level of IL-18 in the BALF of TG rats was lower compared with that of CG rats at T1, T2 and T3. ^*^P<0.001, vs. TG rats. BALF, bronchialalveolar lavage fluid; ELISA, enzyme-linked immunosorbent assay; IL-18, interleukin 18; T1, 30 min after ligating; T2, 30 min after reperfusion; T3, 60 min after reperfusion; TG, treatment group; CG, control group.

ELISA was used to measure the level of IL-18 in peripheral blood and BALF. The results demonstrated that the level of IL-18 in TG rats was lower in peripheral blood and BALF (Figs. 6 and 7). The results indicate that breviscapine reduces the inflammatory reaction.

## Discussion

IL-18 is mainly released from activated pulmonary macrophages. As a pro-inflammatory cytokine, IL-18 promotes T-cell maturation, enhances neutrophil activity and induces the production of inflammatory mediators, including TNF-α, IL-1β and IL-8. In addition to this, IL-18 promotes the expression of ICAM-1 (7–9).

The ICAM-1 protein is mainly located on the surface of endothelial cells and is barely expressed on the majority of human tissues under physiological conditions (10,11). The level of expression of ICAM-1 on endothelial cells is increased under pathological conditions. ICAM-1 interacts closely with integrin located on the surface of granulocyte cells, which causes aggregation, adhesion and the release of leucocytes, as well as immediate cytokine release (12,13). This process is the molecular biological basis of the inflammatory reaction.

Based on this, we established a rat model of acute lung injury induced by left heart ischemic reperfusion and treated the model rats with breviscapine. Then, we used various methods to determine the effect of breviscapine on lung function. We observed the respiration curve, measured PaO_2_ levels, determined the expression of IL-18 and ICAM-1 and detected the levels of IL-18 in the peripheral blood and BALF.

The results demonstrated that respiration was better in amplitude and duration time in the TG rats compared with that of the TG rats at the same time-point (Figs. 1 and 2). The PaO_2_ in the TG was higher compared with that in the CG (Fig. 3). These results indicate that breviscapine may protect respiratory function.

To further explore the possible mechanism by which breviscapine protects respiratory function, we measured the expression levels of IL-18 and ICAM-1 in lung tissue by immunohistochemistry and analyzed the levels of IL-18 in the peripheral blood and BALF by ELISA. The results revealed that the levels of IL-18 in the peripheral blood and BALF of the TG rats were clearly lower compared with those of the CG rats (Figs. 6 and 7), and the expression levels of IL-18 and ICAM-1 in the TG rats were markedly reduced (Tables I and II, Figs. 4 and 5). These results indicate that breviscapine inhibits the expression of IL-18 and ICAM-1 in lung tissue. To determine whether the expression of IL-18 also affects the expression of ICAM-1, we analyzed the correlation between IL-18 and ICAM-1 expression. Analysis of the data revealed that the expression of IL-18 had a positive correlation with the expression of ICAM-1 in the same lung tissue of TG rats, and that low IL-18 expression levels always coincided with low ICAM-1 expression levels (Table III). These results indicate that breviscapine reduces the release of IL-18 by lowering the aggregation and adhesion of neutrophils. As a result, the release of ICAM-1 is reduced and the inflammatory reaction is weakened. Therefore, breviscapine may decrease the acute lung injury induced by left heart ischemic reperfusion by inhibiting the inflammatory reaction.

In conclusion, in the acute lung injury model induced by left heart ischemic reperfusion, breviscapine was able to decrease the expression of IL-18 and ICAM-1, and relieve inflammatory injury in the lungs, therefore, lung function was protected.

## Figures and Tables

**Table I.** Comparison of the expression level of IL-18 in lung tissue between the two groups.

Time	n	IL-18				Z	P-value
		−	+	++	+++		
T1							
TG	10	4	3	3	0		
CG	10	1	4	3	2	1.972	0.037
T2							
TG	10	2	3	3	1		
CG	10	1	1	5	3	1.963	0.042
T3							
TG	10	2	2	4	2		
CG	10	0	1	4	5	2.489	0.023

T1, 30 min after ligating; T2, 30 min after reperfusion; T3, 60 min after reperfusion; TG, treatment group; CG, control group; IL-18, interleukin 18.

**Table II.** Comparison of the expression of ICAM-1 in lung tissue between the two groups.

Time	n	ICAM-1				Z	P-value
		−	+	++	+++		
T1							
TG	10	3	3	3	1		
CG	10	3	4	2	1	2.158	0.014
T2							
TG	10	2	3	2	2		
CG	10	1	4	3	2	1.968	0.043
T3							
TG	10	2	3	3	2		
CG	10	1	1	4	4	2.263	0.012

ICAM-1, inflammatory cell adhesion molecule-1; T1, 30 min after ligaturing; T2, 30 min after reperfusion; T3, 60 min after reperfusion; TG, treatment group; CG, control group.

**Table III.** Correlation between IL-18 and ICAM-1 expression in the lung tissue of the TG.

ICAM-1	IL-18			
	−	+	++	+++
T1				
−	0	0	0	0
+	0	1	2	0
++	0	1	2	1
+++	0	0	2	1
T2				
−	0	1	0	0
+	0	1	2	0
++	0	0	2	1
+++	0	1	1	1
T3				
−	0	0	0	0
+	0	1	1	0
++	0	1	2	1
+++	0	0	2	2

Rank correlation coefficient anaylsis revealed that the expression of IL-18 had a positive correlation with the expression of ICAM-1 in the lung tissue of the TG (T1: r=0.612, P=0.001; T2: r=0.524, P=0.001; T3: r=0.833, P=0.012). ICAM-1, inflammatory cell adhesion molecule-1; IL, interleukin; T1, 30 min after ligating; T2, 30 min after reperfusion; T3, 60 min after reperfusion; TG, treatment group.

## Abbreviations:

IL-18: interleukin 18;
ICAM-1: inflammatory cell adhesion molecule-1;
TG: treatment group;
CG: control group;
T1: 30 min after ligaturing;
T2: 30 min after reperfusion;
T3: 60 min after reperfusion;
PaO_2_: arterial partial pressure of oxygen;
BALF: bronchialalveolar lavage fluid;
PCT: procalcitonin;
NE: neutrophil elastase;
ELISA: enzyme-linked immunosorbent assay;
ANOVA: analysis of variance.
